# Forecast and analysis of the cosmological redshift drift

**DOI:** 10.1140/epjc/s10052-017-5479-0

**Published:** 2018-01-08

**Authors:** Ruth Lazkoz, Iker Leanizbarrutia, Vincenzo Salzano

**Affiliations:** 1 Department of Theoretical Physics, University of the Basque Country UPV/EHU, P.O. Box 644, Bilbao, 48080 Spain; 2 Institute of Physics, University of Szczecin, Wielkopolska 15, 70-451 Sczcecin, Poland

## Abstract

The cosmological redshift drift could lead to the next step in high-precision cosmic geometric observations, becoming a direct and irrefutable test for cosmic acceleration. In order to test the viability and possible properties of this effect, also called Sandage–Loeb (SL) test, we generate a model-independent mock data set in order to compare its constraining power with that of the future mock data sets of Type Ia Supernovae (SNe) and Baryon Acoustic Oscillations (BAO). The performance of those data sets is analyzed by testing several cosmological models with the Markov chain Monte Carlo (MCMC) method, both independently as well as combining all data sets. Final results show that, in general, SL data sets allow for remarkable constraints on the matter density parameter today $\varOmega _m$ on every tested model, showing also a great complementarity with SNe and BAO data regarding dark energy parameters.

## Introduction

Within the general relativity (GR) framework no reliable explanation to the current acceleration of the universe exists which is simpler than a $\varLambda$-term or cosmological constant [1]. It behaves as a fluid with negative pressure [2], thus driving gravitational repulsion. This is of course also the kind of behavior displayed by the plethora of other possible fluids so far proposed to try to accommodate data better than a cosmological constant. In broad terms, these settings, causing the universe to accelerate, are usually included in the so-called dark energy theories (for reviews, see [3–7]). There are other theoretical routes with different levels of complexity (not necessarily unrelated [8]) which venture to modify GR.

The background expansion of the universe can be measured with a lot of different probes: luminosity distances from Type Ia Supernovae (SNe) [9–12]; the acoustic peaks in the Cosmic Microwave Background (CMB) [13, 14]; their counterpart imprinted in clustered matter, i.e. Baryon Acoustic Oscillations (BAO) [15–19]; through the matter power spectrum obtained from weak lensing [20, 21]. Usually a time integral along redshift connects those data with the expansion rate/history of the universe, the Hubble parameter *H*(*z*). It is enough to constrain the geometry and energy content of the universe quite satisfactorily.

On the other hand it is expected that the expansion of the universe will make the redshift of a given astrophysical object exhibit a drift over time, which should in principle be amenable to giving an accurate description of that very same expansion once an underlying model is chosen. While looking for a possible temporal variation of the redshift of extra-galactic sources, Sandage came in 1962 [22] to the conclusion that it should indeed occur. However, the limited technological resources on deck at that epoch lead to the inference that a measurement time interval of the order of $10^7$ years would be required for a signal detection. When new spectroscopic techniques became available to astrophysicists, Loeb considered the concept [23] anew in 1998, he concluded that the new technology would allow a reduction in the observation time interval of a few decades. This cosmological redshift drift measurement, also called the Sandage–Loeb (SL) test, would then provide a direct proof of the accelerated expansion of the universe. In fact, this temporal variation is directly related to the expansion rate at the source redshift, being thus a direct measurement for the Hubble function.

The last results of the *Planck* survey [13] have made us enter an ultra high-precision cosmology era. Other future surveys are scheduled which should further improve the accuracy of cosmological measurements, for example *Euclid* [24], *Wide-Field Infrared Survey Telescope (W-First)* [25] or Square Kilometer Array (SKA) [26].

Thus, in the near future available resources will allow us to start thinking about the next level of cosmological observational data to which the cosmological redshift drift will contribute, complementing the previously cited surveys. However, even with future precision radio telescopes, the measurement of the SL effect represents a difficult enterprise [27] as it demands several years of observation (usually some decades) to register enough signal-to-noise ratio so as to yield a possible reliable detection of the cosmological redshift drift signal. Best candidate objects for a feasible detection of this faint signal are good Hubble flow tracers as far as possible [28]. As put forward by Loeb [23], an auspicious target would be the Lyman-$\alpha$ forest measurements of distant quasars (QSO). With the spectroscopic techniques that are operational in the near future, like the CODEX (COsmic Dynamics and EXo-earth experiment) experiment [29] proposed for the European-Extremely Large Telescope (E-ELT) or radio telescopes as SKA [30], these observations will grant access to direct measurements of the Hubble parameter up to redshifts $\sim 5$, a so far not yet observed redshift range. Thus, the SL test will open a new “cosmological window”.

Due to near future possibilities to measure the cosmological redshift drift, this type of observations recently has drawn some attention. The reconstruction of the theoretical SL signal that different cosmological models would produce has been explored quite extensively [31, 32]. It turns out that the range and variety of the different cosmological redshift drift signals created by various models is remarkable: from those created by different proposals for dark energy’s equation of state or modified gravity [33, 34] to the ones created by backreaction in an inhomogeneous universe without the presence of dark energy [35]; from the peculiar signals for Lemaître–Tolman–Bondi models [36, 37] to even a null signal [38] for the $R_{h}=c t$ Universe, or other several exotic scenarios [39–44]. SL signals have been used as a hypothetical geometric cosmic discriminant [45–47] to show the corresponding improvement in the constraints that can be achieved due to the degeneracy breaking (around $20\%$ of improvements for dark energy parameters and even $65\%$ for matter density). SL mock data sets have been applied with similar results as cosmic observational discriminators to test other various models, like interactive dark energy models [48, 49], modified gravity [50, 51] and other exotic cosmologies [52, 53]. Their power to differentiate models has been exploited also in the context of the model-independent approach of cosmography [54, 55]. Besides, some new approaches [56] can lead to ambitious ideas, such as real-time cosmology [57].

We stress again the fact that the measurement of the cosmological redshift drift is not an easy pursuit. It requires a lot of planning due to the large observation time interval of the survey. Thus, foreseeing the contribution and behavior of this type of measurements is important we precisely carry out a quite thorough forecast analysis of cosmological redshift drift constraints on various cosmological models. The analysis includes a comparison between the proposed SL data with other future planned surveys, generating mock data based on the given specifications. Furthermore, unlike previous work, all mock data sets are generated in a fully model-independent way, without choosing a fiducial cosmological model to generate the points. In Sect. 2 the mathematical formalism of the cosmological redshift drift is introduced and the details of the mock data sets we use for our predictions are given. We find it convenient to produce a SL data set but also use auxiliary SNe and BAO data. In Sect. 3, we explain our MCMC procedure which will eventually constrain the cosmological models we have chosen as reference. Finally, in Sect. 4, the outcomes of the statistical analysis are presented and discussed. Then the main conclusions are summarized.

## Cosmological redshift drift

A preliminary straightforward calculation introduces the main observable quantity we are going to focus on, i.e. the cosmological redshift drift, (see for example [29] or [33]). In a homogeneous and isotropic universe with a Friedmann–Robertson–Walker metric a source at rest emitting electromagnetic waves isotropically, without any (significant) peculiar velocity, is considered. Thus, the comoving distance between the source and an observer can be considered fixed. If the source emits electromagnetic waves during time $(t_e, t_e+\delta t_e)$ and these are detected by the observer in the interval $(t_o, t_o+ \delta t_o)$, where $t_e$ is the emission time and $t_o$ is the time they reach the observer, the following relation is satisfied

1 $$\begin{aligned} \int _{t_e}^{t_o} \frac{\mathrm{d}t}{a(t)} = \int _{t_e+\delta t_e}^{t_o+\delta t_o} \frac{\mathrm{d}t}{a(t)}, \end{aligned}$$

provided the universe through which the waves travel is a spatially flat Friedmann–Robertson–Walker spacetime. If the time intervals are small $(\delta t_e, \delta t_o \ll t_e, t_o)$ the above expression leads to the well-known redshift relation between the emitted and the observed radiation

2 $$\begin{aligned} \frac{\delta t_e}{a(t_{e})} = \frac{\delta t_o}{a(t_o)} \Rightarrow \frac{\lambda _o}{\lambda _e} = \frac{a(t_o)}{a(t_e)} = 1+z_e(t_o), \end{aligned}$$

where $z_{e}(t)$ is the redshift of the source as measured at a certain observation time $t_o$. Other waves can be emitted by the source $\delta t_e$ time later, specifically at time $t_e + \delta t_e$. They will be observed at $t_o + \delta t_o$. Concerning these waves Eq. (2) has to be modified regarding the new time periods and redshift. Thus, the observer can measure the difference between the redshifts observed at $t_o$ and $t_o +\delta _o$:

3 $$\begin{aligned} \varDelta z_e=z_e(t_o+\delta t_o)-z_e(t_o)=\frac{a(t_o+\delta t_o)}{a(t_e+\delta t_e)}-\frac{a(t_o)}{a(t_e)} \; . \end{aligned}$$

Within the $\delta t / t \ll 1$ approximation, the first ratio can be expanded to linear order

4 $$\begin{aligned} \frac{a(t_o+\delta t_o)}{a(t_e+\delta t_e)} \simeq \frac{a(t_o)}{a(t_e)} + \frac{\dot{a}(t_o) \delta t_o}{a(t_e)} - \frac{a(t_o) \dot{a}(t_e) \delta t_e}{a(t_e)^2} \; . \end{aligned}$$

Inserting Eq. 2 into the first order expansion in Eq. 4 an approximated expression for the redshift variation can be obtained,

5 $$\begin{aligned} \varDelta z_e \simeq \left[ \frac{\dot{a}(t_o)-\dot{a}(t_e)}{a(t_e)}\right] \delta t_o. \end{aligned}$$

Under the assumption that the observation time is today, we normalize by letting the corresponding scale factor satisfy $a(t_o)=1$. Then, using both the Friedmann equation and the known redshift equation Eq. (2), the above expression can be rewritten in terms of the Hubble parameter $H(z)=\dot{a}(z)/a(z)$

6 $$\begin{aligned} \varDelta z_e = \delta t_o \left[ H_0(1+z_e) - H(z_e) \right] , \end{aligned}$$

with $H_0=H(z_0)$ being the Hubble constant today. This redshift variation can be expressed as a spectroscopic velocity shift $\varDelta v \equiv c \varDelta z_e /(1+z_e)$. Using the dimensionless Hubble parameter $E(z)=H(z)/H_0$, the final expression can be found:

7 $$\begin{aligned} \varDelta v = c H_0 \delta t_o \left[ 1 - \frac{E(z_e)}{1+z_e} \right] \; . \end{aligned}$$

### Sandage–Loeb mock data set

In order to generate our SL observational mock data set in a fully model-independent manner, we try to derive a Hubble function from a phenomenological distance modulus, in a fashion similar to [58]. We propose this observable because it is well measured by Type Ia Supernovae (SNe) and can be extended to high redshifts, even if with lower precision, by Gamma Ray Bursts (GRBs, Mayflower sample) [59]. We model this phenomenological distance modulus thus:

8 $$\begin{aligned} \mu _\mathrm{fit}(z) = a + 5 \log _{10} \left[ F_\mathrm{fit}(z;b,c,d,e) \right] , \end{aligned}$$

where $F_\mathrm{fit}$ is an ad hoc proposed function (among many) mimicking the luminosity distance. This phenomenological function is then fitted using the SNe data set Union 2.1 [11] for the low-redshift regime and the GRBs sample calibrated by the Padé method [59] for the high-redshift one. Once $\mu _\mathrm{fit}$ is fitted, other observational quantities relevant to our work can easily be obtained. For instance, the Hubble function can be derived recalling the relation

9 $$\begin{aligned} \mu (z) = 5 \log _{10} \mathrm{d}_L(z) + \mu _{0}, \end{aligned}$$

where, in the spatially flat universe we are considering, the dimensionless luminosity distance $\mathrm{d}_L$ is defined as

10 $$\begin{aligned} \mathrm{d}_L(z) = (1+z) \int _0^z \frac{\mathrm{d}z'}{E(z')}, \end{aligned}$$

and $\mu _0$ stores all the information related to the constants involved, such as the speed of light *c*, the Hubble constant $H_0$ and the SNe absolute magnitude. By comparing both distance moduli, $\mu$ from Eq. (9) and $\mu _\mathrm{fit}$ from Eq. (8), one realizes that the dimensionless luminosity distance $d_L(z)$ is equivalent to the function $F_\mathrm{fit}$. Thus, the dimensionless Hubble function is

11 $$\begin{aligned} E_\mathrm{fit}(z)= \left( \frac{\mathrm{d}}{\mathrm{d}z} \frac{F_\mathrm{fit}(z;b,c,d,e)}{(1+z)} \right) ^{-1}. \end{aligned}$$

**Fig. 1:**
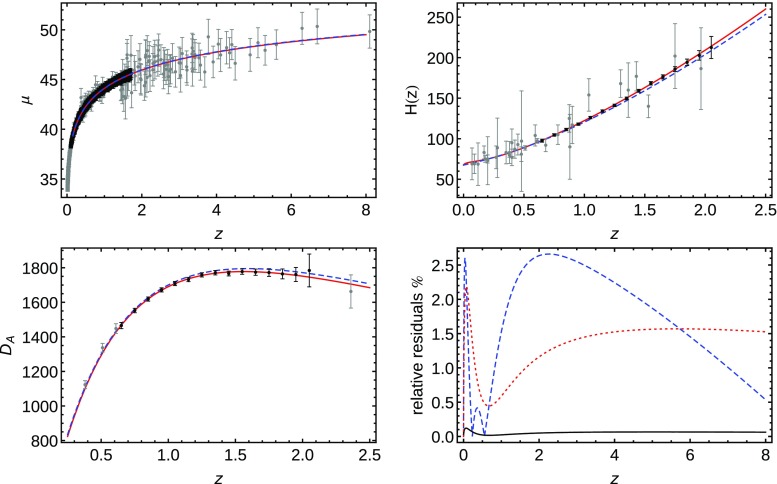
Top left panel: Comparison between the selected phenomenological function $F_\mathrm{fit}(z;b,c,d,e)$ given in Eq. (12) (solid red) with the *Planck* $\varLambda$CDM (dashed blue) described in the text. Gray dots and bars are distance modulus values and related errors for SNe Union and Mayflower GRBs samples and black ones are for our generated SNe mock data. Top right panel: comparison between the *H*(*z*) function derived from Eq. (12) (solid red) with that corresponding to the *Planck* $\varLambda$CDM (dashed blue) described in the text. Gray dots and bars are expansion rate values and related errors from cosmic chronometers and black ones our generated mock data. Bottom left panel: comparison of the $D_A(z)$ function derived from Eq. (12) (solid red) with that corresponding to the *Planck* $\varLambda$CDM (dashed blue) case described in the text. Gray dots and bars are angular diameter distances values and related errors from BOSS and SDSS and black ones our generated mock data. Bottom right panel: relative residuals between our model and the *Planck* $\varLambda$CDM for the Hubble function (dashed blue), the distance modulus (solid black) and the angular diameter distance (dotted red)

Once such a phenomenological dimensionless Hubble parameter $E_\mathrm{fit}(z)$ is obtained, we can “mimick” all the cosmological probes we need for our analysis as they are all related to it. In this way cosmological-model-independent mock data sets can be created where the only intrinsic information that is used for $E_\mathrm{fit}$ is that it has to be able to fit present data (in this case, SNe and GRBs). Of course, some arbitrariness lies behind the choice of the phenomenological function $F_\mathrm{fit}$; we have tried to use the most general type of functions possible and we have selected the best one based on a simple best-fitting (minimum $\chi ^2$) criterion. The best performing function we have found is

12 $$\begin{aligned} F_\mathrm{fit}(z;b,c,d,e) = \frac{z (1 + b \log [1 + z]^{d})}{(1 + c \log [1 + z]^{e})}, \end{aligned}$$

where the values for the parameters are shown in Table 1. It can be seen in Fig. 1, in the top left panel, that this function fits the distance modulus points of the Union 2.1 [11] and Mayflower [59] data sets as much satisfactorily as a $\varLambda$CDM with *Planck* values, $\varOmega _{m}=0.3121$ (sixth column of Table 4 in [13]). In the top right panel, we also compare the expansion rate function *H* which can be derived from Eq. (12) with the same *Planck* $\varLambda$CDM and with data from cosmic chronometers [60]. In the bottom left panel, the comparison between angular diameter distance derived from Eq. (12) and the same *Planck* $\varLambda$CDM is done, with the data coming as comoving angular diameter distance from galaxy clustering (BAO+FS column of Table 7 in [61]) and physical angular diameter distance coming from quasar cross-correlation (Eq. (21) in [18]). Finally, in the bottom right panel, it can be seen that the difference between our model and the *Planck* $\varLambda$CDM is minimal for the case of the distance modulus $(\sim 0.1\%)$ and small for both the Hubble function $(\sim 2.5\%)$ and the angular diameter distance $(\sim 2\%)$, all over the redshift range we cover with our mock data in our analysis.

**Table 1:** Parameter values of $F_\mathrm{fit}$

	Estimate	Standard error
*a*	43.2025	0.146659
*b*	2.29876	1.60875
*c*	0.92048	0.969826
*d*	1.05317	0.62311
*e*	0.814751	0.922533

Once we have our $E_\mathrm{fit}(z)$, we only need to specify a fiducial value for the Hubble constant to insert in Eq. (7), whose effect is only the rescaling of the velocity shift value. We fix the value of $H_0= 67.51 \, \mathrm{km/s/Mpc}$ from the TT, TE, EE + lowP + lensing baseline model of *Planck* [13]. Then, concerning the SL data, the points lie in the redshift range $2< z < 5$, randomly distributed within the following bins: $2<z<3$ (13 points), $3<z<3.5$ (7 points), $3.5<z<4$ (4 points), $4<z<4.5$ (3 points) and $4.5<z<5$ (3 points). In this way we try to mimic the reduction of the number of data points while increasing the redshift as in [62].

**Fig. 2:**
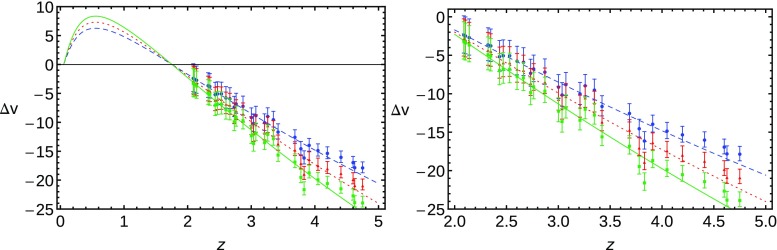
Datasets for SL test based on $F_\mathrm{fit}(z;b,c,d,e)$ for different observation periods: blue (circle and dashed line) for 24 years, red (triangle and dotted line) for 28 years and green (square and solid line) for 32 years

According to Monte Carlo simulations carried out to eventually mimic results from CODEX [29, 63], the standard deviation on the measured spectroscopic velocity shift $\varDelta v$ can be estimated

13 $$\begin{aligned} \sigma _{\varDelta v} = 1.35 \frac{2370}{S/N} \sqrt{ \frac{30}{N_\mathrm{QSO}}} \left( \frac{5}{1+z_\mathrm{QSO}} \right) ^x \mathrm{cm \, s^{-1}}, \end{aligned}$$

where *x* is 1.7 for $z\le 4$, and 0.9 beyond that redshift, *S* / *N* is the spectral signal-to-noise ratio of Ly-$\alpha$, $N_\mathrm{QSO}$ is the number of observed quasars and $z_\mathrm{QSO}$ their redshift. The error for the mock data is given by assuming a fixed number of integration time hours which yields a value of $S/N= 3000$ for the signal-to-noise ratio and $N_\mathrm{QSO}=30$ for the number of quasars observed [28]. We also introduce some noise to disperse the data points around the fiducial value derived from $E_\mathrm{fit}$, using a Gaussian distribution centered on such values, and with a standard deviation corresponding to the expected error on the SL observation, $\sigma _{\varDelta v}$, obtained by error propagation from the fitted parameters of the selected function Eq. (12).

Note that the magnitude of the observed cosmological redshift drift is proportional to the observation period although the error does not depend on it. Thus, once a data set for some given observational time $\varDelta t_A$ is created, any new mock data set with different observation period $\varDelta t_B$ can easily be calculated by

14 $$\begin{aligned} \varDelta v_B = \frac{\varDelta t_B}{\varDelta t_A} \varDelta v_A. \end{aligned}$$

We use the three observation periods of 24, 28 and 32 years, which are the most illustrative among the data sets tested. The resulting data sets for SL test can be seen on Fig. 2.

### Auxiliary mock datasets

Additional future mock data sets are included alongside the cosmological redshift drift data set in order to better constrain models. Basically, the reason why we introduce in the picture these other probes is our interest in studying and quantifying the relative performance of SL with respect to more standard and used probes and our aim of finding out whether the cosmological redshift drift data have some degree of complementarity with them, thus providing eventual tighter constraints. These auxiliary mock data sets are created from the same model-independent function of Eq. (8).

#### *W-First* SNe

The first mock data set we produce is a SNe catalog based on the *W-First* forecast [25] which includes 2725 SNe randomly picked in redshift bins of $\delta z = 0.1$ spread through a redshift range of $0.1< z < 1.5$ according to the distribution given by [25].

Given that in the SNe case one measures the distance modulus, direct use can be made of the fitted function Eq. (8) to generate the mock data points. As in the SL case some Gaussian noise is introduced to disperse the data points around the mean value.

To create the error bars for this catalog and the dispersion for the Gaussian noise the information given in [25] is used. The statistical errors they account for are the following: the photometric measurement error, $\sigma _\mathrm{meas}=0.8$; the intrinsic luminosity dispersion $\sigma _\mathrm{int}=0.08$; and the gravitational lensing magnification $\sigma _\mathrm{lens}=0.07$. Besides, they assume a systematic error $\sigma _\mathrm{sys}=0.01 (1+z)/1.8$. Thus the total error per SNe is

15 $$\begin{aligned} \sigma _\mathrm{tot}=\sqrt{ \sigma _\mathrm{stat}^2 + N_\mathrm{SN }\sigma _\mathrm{sys}^2 }, \end{aligned}$$

where $\sigma _\mathrm{stat}=\sqrt{\sigma _\mathrm{meas}^2+\sigma _\mathrm{int}^2+\sigma _\mathrm{lens}^2}$ and $N_\mathrm{SN}$ is the number of SNe in the bin. The data set generated for the *W-First* SNe survey is shown on Fig. 1.

#### Euclid BAO

The second data set considered is BAO. We choose the future *Euclid* survey [24] as the experiment to reproduce. The two quantities considered are the angular diameter distance

16 $$\begin{aligned} D_A(z) = \frac{c}{1+z} \int _0^z \frac{\mathrm{d}z'}{H(z')}, \end{aligned}$$

normalized by the sound horizon $D_A(z)/r_s$, and the Hubble parameter times the sound horizon, $H(z) \, r_s$, where the value of $r_s=144.71$ *Mpc*, consistent with the previous $H_{0}$, is used [13].

Both the angular diameter distance and the Hubble parameter are reconstructed, again using Eq. (8). It is already discussed that the Hubble parameter can be inferred as in Eq. (11), once a value for $H_{0}$ is decided upon. Instead, in order to derive the angular diameter distance from Eq. (8), its definition and its relation with the luminosity distance $(1+z)^2 D_A=D_L$ are used, thus leading to

17 $$\begin{aligned} D_A(z) = \frac{c}{H_0} \frac{F_\mathrm{fit}(z;b,c,d,e)}{(1+z)^2} \; . \end{aligned}$$

The redshift values of the data set are taken from [64]. They specifically are the central redshifts of 15 bins with $\delta z =0.1$ width, spread from $z=0.5$ to $z=2.1$. The error in each redshift value for both $D_A$ and $H_0$ is build from the percentage error also given in [64]. Finally, some Gaussian noise is introduced using the error from each bin as dispersion when generating the points $D_A(z)/r_s$ and $H(z) \, r_s$. The resulting data sets can be seen in Fig. 1 before normalizing the observables by the comoving sound horizon $r_s$.

## Testing models

Within the Bayesian framework, we aim to find how SL constrains the probability distribution function of some cosmological parameters. For that purpose the posterior distribution is needed, or equivalently the likelihood, which can be straightforwardly computed with MCMC sampling while minimizing the $\chi ^2$ function. The knowledge of the posterior probability gives a better and more complete information as regards the parameters, including the full correlation among them.

Thus, once we have the mock data sets, we build the $\chi ^2$ function for each observable and, once all contributions are summed, we minimize the total $\chi ^2$ in order to perform our statistical analysis. The $\chi ^2$ contribution for the spectroscopic velocity shift is simply

18 $$\begin{aligned} \chi ^2_{SL} = \sum _i \left( \frac{ \varDelta v^{theo}_i - \varDelta v_i ^\mathrm{mock}}{\sigma _{\varDelta v_i}} \right) ^2, \end{aligned}$$

where $\varDelta v^{theo}_{i} = \varDelta v(z_i)$ follows from Eq. (7). The errors $\sigma _{\varDelta v_i}$ are given by Eq. (13). The errors are arranged into a diagonal covariance matrix. Depending on whether the SL surveys will use overlapping redshift bins or not, the error could be more realistically given by a non-diagonal covariance matrix. As we lack such information, we adopt the optimistic diagonal covariance matrix assumption, always keeping in mind that it could lead to a general underestimation of the global errors on the cosmological parameters. The period of observation $\varDelta t_o$ as specified above changes depending on the mock SL survey tested. In the case of the $\chi ^2$ contribution of SNe $\chi ^2$ we have

19 $$\begin{aligned} \chi ^2_\mathrm{SN}= \sum _i \frac{\left( \mu (z_i) - \mu ^\mathrm{mock}_i\right) ^2}{\sigma _{\mu _i}^2}\ , \end{aligned}$$

where the error is given by Eq. (15). We can marginalize $\chi ^2$ over the parameter $\mu _0$ by expanding the $\chi ^2$ in Eq. (19) with respect to $\mu _0$ as

20 $$\begin{aligned} \chi ^2_\mathrm{SN}= A - 2 \mu _0 B + \mu _0^2 C\ , \end{aligned}$$

where

21 $$\begin{aligned} A= & {} \sum _i \frac{\left( \tilde{\mu }(z_i) - \mu ^\mathrm{mock}_i\right) ^2}{\sigma _{\mu _i}^2}\, , \nonumber \\ B= & {} \sum _i \frac{ \tilde{\mu }(z_i) - \mu ^\mathrm{mock}_i }{\sigma _{\mu _i}^2}\, , \nonumber \\ C= & {} \sum _i \frac{1}{\sigma _{\mu _i}^2 } \; . \end{aligned}$$

Then, integrating $\mu _0$ out of the likelihood $\mathcal{L}= e^{- \frac{\chi ^2_\mathrm{SN}}{2}}$ we can retrieve

22 $$\begin{aligned} \tilde{\chi }^2_\mathrm{SN}=A- \frac{B^2}{C} + \ln \frac{C}{2 \pi } \, , \end{aligned}$$

where $\tilde{\chi }^2_\mathrm{SN}$ has now no dependence on the $\mu _0$ parameter. We have to point out that also in this case we are using a diagonal covariance matrix because it is not possible to forecast out-of-diagonal terms. This may lead to underestimated errors on the cosmological parameters. With BAO we have two correlated measurements to contribute to the total $\chi ^2$; these are $H(z) \, r_s$ and $D_A(z)/r_s$. With the Hubble parameter from our phenomenological fit and the angular diameter defined in the previous section, the comoving sound horizon $r_s$ reads

23 $$\begin{aligned} r_s(z_*) = \frac{1}{H_0} \int _{z_*}^\infty \mathrm{d}z'\frac{c_s}{E(z')} = \frac{1}{H_0} \int _0^{a_*} \frac{\mathrm{d}a'}{a'^2} \frac{c_s}{E(a')} \end{aligned}$$

where the sound speed is $c_{s} = c /\sqrt{3(1+ \overline{R_b} a)}$, with $\overline{R_b}=31500 \varOmega _b h^2 (T_{CMB}/2.7K)^{-4}$ and $T_{CMB}=2.725$ [65]. The comoving sound horizon $r_s(z_*)$ is evaluated at photon-decoupling epoch redshift given by the fitting formula [66]

24 $$\begin{aligned} z_*= & {} 1048 \left[ 1+0.00124 (\varOmega _b h^2)^{-0.738} \right] \nonumber \\&\quad \times \left[ 1+b_1 (\varOmega _m h^2)^{b_2} \right] \; , \end{aligned}$$

with

25 $$\begin{aligned} b_1= & {} 0.0783 (\varOmega _b h^2)^{-0.238} \left[ 1+ 39.5 (\varOmega _b h^2)^{0.763} \right] ^{-1}, \; \; \end{aligned}$$

26 $$\begin{aligned} b_2= & {} 0.560 \left[ 1+ 21.1 (\varOmega _b h^2)^{1.81} \right] ^{-1} \; , \end{aligned}$$

where $\varOmega _b$ and $\varOmega _m$ are the baryon and matter content of the universe and $h=H_0/100$. The BAO contribution is independently calculated for each redshift, $\chi ^2_\mathrm{BAO}= \sum _i \chi ^2_{BAO_i}$. However, taking into account the correlation of the magnitudes, each term at each redshift has the form

27 $$\begin{aligned} \chi ^2_{BAO_i} = \frac{1}{1-r^2} \left( \frac{\tilde{H}^2_{i}}{\sigma _{\tilde{H}_i}^2} + \frac{\tilde{D}^2_{i}}{\sigma _{\tilde{D}_i}^2} - 2 r \frac{\tilde{H}_{i}}{\sigma _{\tilde{H}_i}} \frac{\tilde{D}_{i}}{\sigma _{\tilde{D}_i}} \right) \, , \end{aligned}$$

where $\tilde{H}_i$ and $\tilde{D}_i$ are the differences between the model predicted and the mock generated measurements,

28 $$\begin{aligned} \tilde{H}_{i}= & {} H(z_i) \, r_s(z_*) - (H\,r_s)^\mathrm{mock}_i , \; \; \end{aligned}$$

29 $$\begin{aligned} \tilde{D}_{i}= & {} \frac{D_A(z_i)}{r_s(z_*)} - \left( \frac{D_A}{r_s} \right) ^\mathrm{mock}_i \, . \end{aligned}$$

The correlation between the two magnitudes $H\,r_s$ and $D_A/r_s$ in each redshift is fixed as $r=0.4$ [67]. Since CMB data are not used, SNe data are marginalized over the parameter $H_0$ and BAO data do not give information about it (because $D_{A}/r_s(z_*)$ and $H\, r_s(z_*)$ do not basically depend on it), the parameters $H_0$ and the combination $\varOmega _b h^2$ cannot be well constrained. Thus, we also include a Gaussian prior for $H_0$ and $\varOmega _b h^2$, with $H_0^{Planck}= 67.51 \pm 0.64$ and for $\varOmega _b h^2_\mathrm{Planck} = 0.02226 \pm 0.00016$ both derived from *Planck* [13].

The minimization of the $\chi ^2$ function was performed using the MCMC method [68–70], with a Wolfram Mathematica self-developed code based on the Metropolis–Hastings algorithm. In order to see the contribution of each mock data set to the total $\chi ^2$ we also have run chains for each data set separately. In this way, the cosmological redshift drift data are compared with those from the other future surveys. Thus it is found whether it will be useful and up to what extent. Moreover, for a round analysis regarding the viability of the Sandage–Loeb test and the performance of the future (mock) surveys several dark energy scenarios are put to the test.

### $\varLambda$CDM

The first model we test is the extremely well-known $\varLambda$CDM model [71, 72], which has no degree of freedom in the dark energy equation of state and whose dimensionless Hubble parameter is given by

30 $$\begin{aligned} E_{\varLambda CDM}^2(a) = \varOmega _m a^{-3}+\varOmega _{r}a^{-4 }+ \varOmega _{\varLambda } \, , \end{aligned}$$

taking $$\varOmega _{\varLambda }=1 - \varOmega _{m} - \varOmega _{r}$$ with [73]

31 $$\begin{aligned} \varOmega _{r}= \varOmega _{m} \left[ 1 + 2.5 \times 10^4 h^2 \varOmega _m (T_{CMB}/2.7)^{-4} \right] ^{-1} \end{aligned}$$

and using $T_{CMB}=2.7255 \, K$ [65]. We enforce $0<\varOmega _m<1$, and $0<\varOmega _b< \varOmega _m<1$ as physical priors. The same is done for all the other models analyzed in this paper. The results of the Bayesian analysis for the $\varLambda$CDM model can be seen in Table 2 and Fig. 4.

### Quiessence

The second model tested is quiessence [74, 75] with a single degree of freedom in the dark energy equation of state parameter (i.e. no redshift dependence). Its dimensionless Hubble parameter is given by

32 $$\begin{aligned} E_{Q}^2(a) = \varOmega _m a^{-3}+\varOmega _{r}a^{-4 } + \varOmega _{\varLambda }a^{-3(1+w)}, \end{aligned}$$

where all the parameters except *w* are built like in the $\varLambda$CDM model and have the same priors. The parameter *w* has the prior $-5<w<0$. This range was chosen after having verified that expanding it further has no influence on results. Table 3 and Fig. 5 show the results for quintessence model.

### Slow-roll dark energy

We consider another one-parameter dark energy model, coming from the slow-roll dark energy scenario described in [76]. Its dimensionless Hubble parameter, taking into account a radiation component [77, 78], is given by

33 $$\begin{aligned} E_{SR}^2(a)= & {} \varOmega _m a^{-3}+\varOmega _{r}a^{-4 } + \nonumber \\&\quad + \varOmega _{\varLambda } \left( \frac{a^{-3}}{\varOmega _m a^{-3} +\varOmega _r a^{-4} + \varOmega _{\varLambda }}\right) ^{(\delta w/\varOmega _{\varLambda })}.\nonumber \\ \end{aligned}$$

For $\delta w$ we impose a prior of the same width as that of the parameter *w* of quiessence. However, as $\delta w$ is supposed to have its mean value at $\delta w=0$, its prior is designed accordingly. Thus, we take $-2.5< \delta w < 2.5$. The results for the slow-roll dark energy model can be found in Table 4 and Fig. 6.

### CPL

We are also interested in testing models of dark energy whose equation of state parameter *w* has more than one degree of freedom. As our first two-parameter dark energy model we take the CPL model [79, 80], its dimensionless Hubble parameter being

34 $$\begin{aligned} E_{CPL}^2(a)= & {} \varOmega _m a^{-3}+\varOmega _{r}a^{-4 } \nonumber \\&\quad + \varOmega _{\varLambda }a^{-3(1+w_0+w_a)} e^{-3 w_a (1-a)}\;, \end{aligned}$$

where all the terms except $w_0$ and $w_a$ are built like in previous models and with the same priors. The parameter $w_0$ has the same prior as *w* does in quiessence; and we take $-5< w_a < 5$ for the second parameter. We demand in this case $w_a + w_0 < 0$ in order to have an equation of state for the DE component which is negative in the asymptotic past. Table 5 and Fig. 7 give the results of our Bayesian analysis for the CPL model.

### Lazkoz–Sendra pivotal dark energy

Another model with two parameters for the equation of state for DE [81] is considered which can be understood easily as a perturbative departure from $\varLambda$CDM up to second order in redshift. Even though it is a different parametrization as compared to CPL it can be also expressed in terms of the parameters $w_0$ and $w_a$ with the same interpretation: $w_0$ is the value of equation of state of the dark energy at present, whereas $w_0+w_a$ is its value in the asymptotic past. Specifically, the Lazkoz–Sendra pivotal dark energy parametrization has the following dimensionless Hubble parameter:

35 $$\begin{aligned} E_\mathrm{LS}^2(a)= & {} \varOmega _m a^{-3} +\varOmega _r a^{-4} +\varOmega _{\varLambda } X(a) \; , \nonumber \\ X(a)= & {} a^{-3(1+w_0+w_a)} e^{\frac{3}{4}(1-a) \left[ 1+w_0-5 w_a + a(w_a-w_0-1) \right] }\nonumber \\ \end{aligned}$$

where all the relative densities $\varOmega _i$ are built like in the CPL case, all parameters also having the same priors as in CPL, including $w_0$ and $w_a$. In case of the Lazkoz–Sendra model the results of the Bayesian analysis are shown in Table 6 and Fig. 8.

## Results and conclusions

In the summary tables for each model are found, the minimum value of $\chi ^2$ is presented and the constraints for all the free parameters and the reduced $\chi ^2_\mathrm{red}$. As explained in previous section the $\chi ^2$-minimization is done using different combinations of data sets. In the tables we first show the results from using BAO and SNe separately and those from joining both. We then move on to present the results from SL only. Finally, the results for the total SNe + BAO + SL combination are presented. When using SL data, each data set with different observation years is treated separately. In this way the performance of the cosmological redshift drift data sets can be clearly analyzed. For each model we also show the confidence contours for the most interesting cosmological parameters. Each MCMC round is tested for statistical convergence using the method described in [82].

In the $\varLambda$CDM scenario we find that the cosmological redshift drift data provide remarkably good constraints on $\varOmega _m$: when those data are used alone we get standard deviations on $\varOmega _m$ which are 2–3 times smaller than those from the SNe+BAO combination. Considering the broad priors taken for $\varOmega _m$ in all cases and the negligible correlation between the Hubble constant *h* and $\varOmega _m$,^1^ we conclude that the result for the matter density $\varOmega _m$ is not influenced by any prior and is solely given by the data.

Indeed, the SL data sets always do better in constraining $\varOmega _m$ than the SNe data and, depending on the model and on the years of observation, even better than the BAO data set. Once we combine the SL data set with the other two, the cosmological redshift drift is still helpful, even though the BAO + SNe data set already greatly improves the constraints in the parameter space. In general, it is clear that the cosmological redshift drift data considerably helps to constrain the parameter $\varOmega _m$ in all the models.

Regarding the dark energy parameters, it can be observed that for most cases the 24 years of observation for SL is not enough to properly constrain them. This can for example clearly be seen from the contours of the parameters $w_0$ and $w_a$ in Figs. 7 and 8. With 28 years SL data, the $1 \sigma$ regions improve noticeable and with 32 years of observation both $1\sigma$ and $2\sigma$ regions are well constrained for all the DE parameters. The best example is seen in Fig. 8. However, similar behavior can be appreciated in the rest of the models. Besides, it is clear that increasing the observation years improves the overall constraining ability of the cosmological redshift drift data sets. It is worth to note that in all these cases the contours of the SL data set are almost perpendicular to the contours of the SNe and BAO data sets, thus showing a great complementarity between SL and the rest of the data sets [83], as for example in the $\varOmega _m$–*w* plane for the quiessence model Fig. 5 or for the dynamical dark energy models Figs. 6, 7 and 8. This is very important because it means that even the cosmological redshift drift data set with the lowest observation period noticeably contributes to improved dark energy insights when used as cosmological probe together with other kind of observations.

**Fig. 3:**
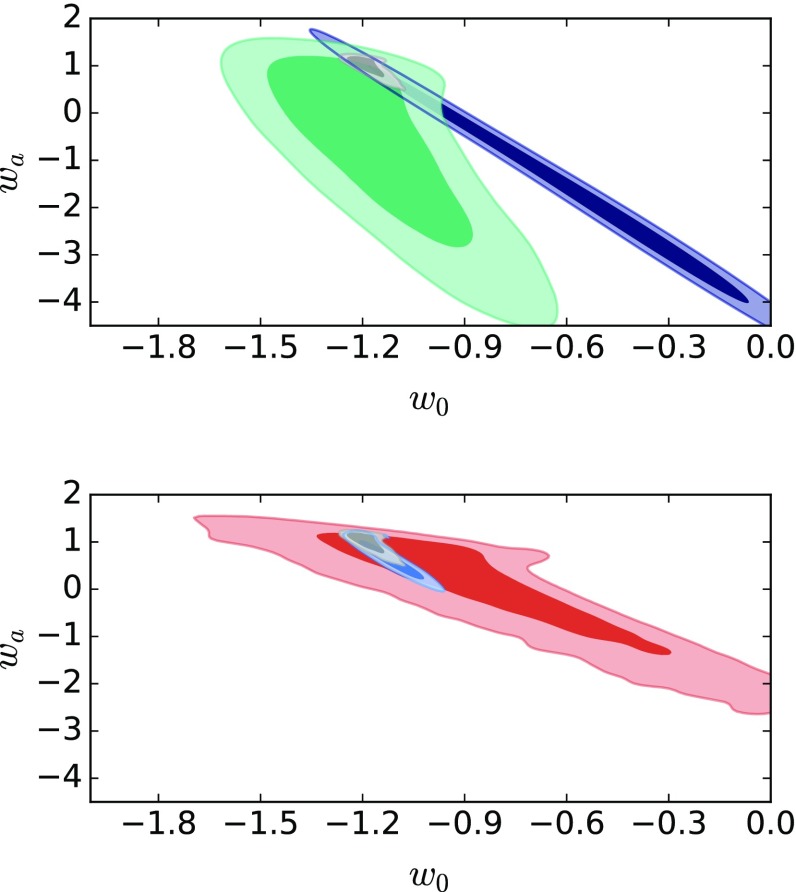
Contours in the $$w_0 $$–$$ w_a$$ plane for CPL; solid contours are for $$1\sigma $$ regions and clear contours are for $$2\sigma $$ regions. Top panel: purple is for the BAO data; green for SNe and gray for SNe + BAO. Bottom panel: red is for 32 years SL data; gray for SNe + BAO; blue for SNe + BAO + SL

However, if one focuses on the $w_0$ and $w_a$ parameters two things can be noted: first, that the best fit for the SNe + BAO case is completely different from the values derived from the SNe and the BAO separate analysis (this is more evident for $w_{a}$ than $w_0$); second, the errors on the $w_0$ and $w_a$ parameters slightly increase when adding cosmological redshift drift data to the SNe+BAO data. Both trends might have an explanation. Concerning the first one, if we look at the top panel of Fig. 3 (this is true for the CPL case but also for the LS model), we can see how unsatisfactorily the SNe and BAO contours overlap: the borders of the $1\sigma$ confidence levels show a small overlap in a region which is far from the best fit expected for each of them when considered separately. This reduces the constraints on the parameters in a considerable way and shifts the best fit estimations (not only in $w_{a}$ but also on $\varOmega _m$). Note also that this behavior is somehow expected and might be counter-productive in the future, as explained in [84]. Anyway, we must also remember we are working with mock data and not real data. Thus the potential future goodness of the joint use of SNe and BAO at present and maybe in the near future is not put at stake. Moreover, we have to remember that in order to gain more insights into a dynamical dark energy model we need to improve the number and the quality of data at high redshift; that is the reason behind pushing SNe observations to higher redshifts [85] for example or employing BAO data at $z\sim 2$. But the strongest hints about the dynamical nature of the dark energy might come from data like SL which are able to cover a larger and deeper redshift range. The second issue discussed above should be exactly connected to this: if we check again the bottom panel of Fig. 3 we can see how the SL data set alone, which should be more sensitive to a dynamical dark energy, determines a consistent shift in the parameter $w_{0}$ with respect to the SNe + BAO case but with smaller uncertainty with respect to both SNe and BAO data separately, which eventually ends in a slightly large error for this parameter for the total SNe + BAO + SL sample.

In the case of models with a single DE parameter whose equation of state is fixed during time high-redshift SL data are also helpful. In the extreme case when SL data are added to the SNe+BAO data set, even the SL data with lowest observational period help constrain the single parameter of DE. However, it is also remarkable how every data set separately constrains the single DE parameter to a different value. Taking into account that the redshift range of each data set is quite different, the fact that they separately have a different value for the parameter could be evidence for a time evolution in the equation of state of DE. This is a clear example of another application for the SL observation, where its high-redshift data could easily test the time evolution of the equation of state of DE once compared to the results of other data sets coming from different sources.

Much of what has been stated above can be easily inferred upon closer examination of the various contours plots. However, these plots are more useful for analyzing the correlation between different parameters. As stated previously, in most of the contour plots a different correlation angle can be seen for the cosmological redshift drift data compared to the other data sets. Thus, it clearly emerges that SL data sets will be of utmost importance in breaking degeneracies among cosmological parameters. Besides, considering the high-redshift data that will be available thanks to cosmological redshift drift we conclude that it can be a cosmic observable worthy to consider.

## Figures and Tables

**Fig. 4:**
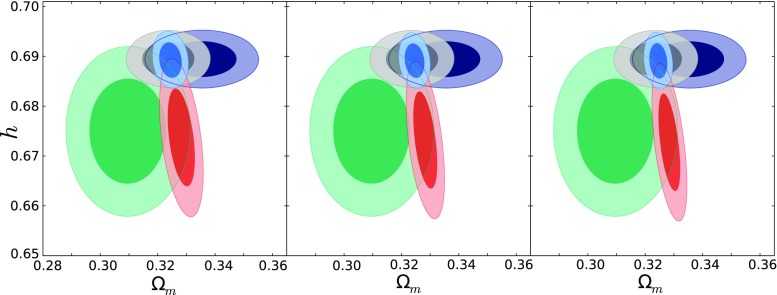
$\varLambda$CDM model; solid contours limit $1\sigma$ regions and clear contours $2\sigma$ region. Purple for the BAO, green for SNe and red for SL data set, gray for SNe + BAO and blue SL + BAO + SNe. First set (left) for 24 years, second (middle) for 28 and third (right) for 32 years

**Fig. 5:**
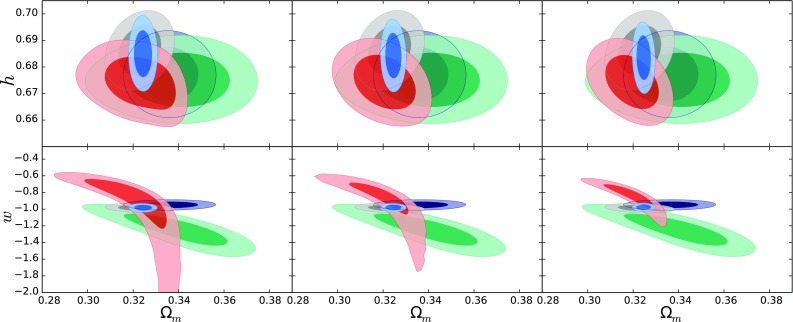
Quiessence model; solid contours limit $1\sigma$ regions and clear contours $2\sigma$ region. Purple for the BAO, green for SNe and red for SL data set, gray for SNe + BAO and blue SL + BAO + SNe. First set (left) for 24 years, second (middle) for 28 and third (right) for 32 years

**Fig. 6:**
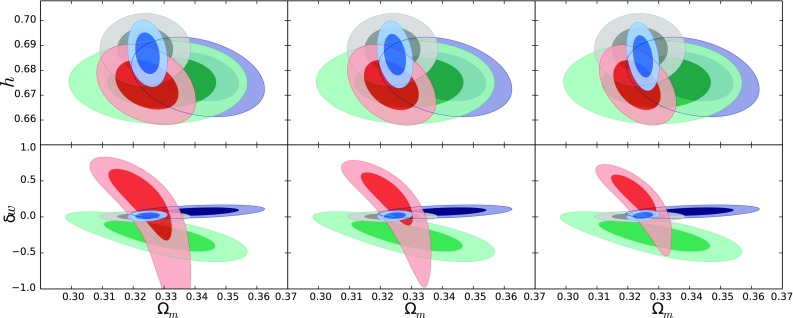
Slow-roll model; solid contours limit $1\sigma$ regions and clear contours $2\sigma$ region. Purple for the BAO, green for SNe and red for SL data set, gray for SNe + BAO and blue SL + BAO + SNe. First set (left) for 24 years, second (middle) for 28 and third (right) for 32 years

**Fig. 7:**
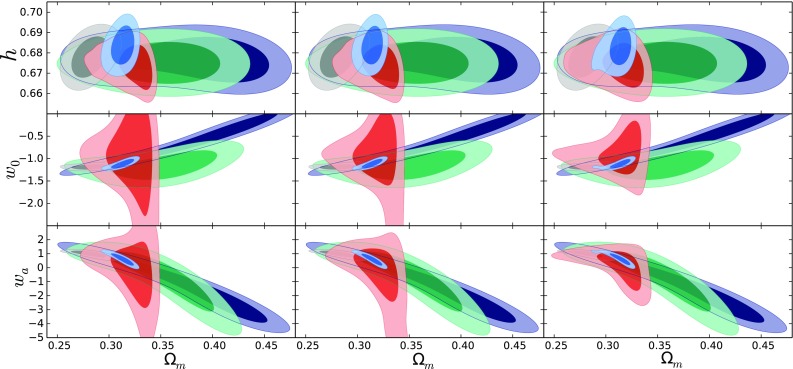
CPL model; solid contours limit $1\sigma$ regions and clear contours $2\sigma$ region. Purple for the BAO, green for SNe and red for SL data set, gray for SNe + BAO and blue SL + BAO + SNe. First set (left) for 24 years, second (middle) for 28 and third (right) for 32 years

**Fig. 8:**
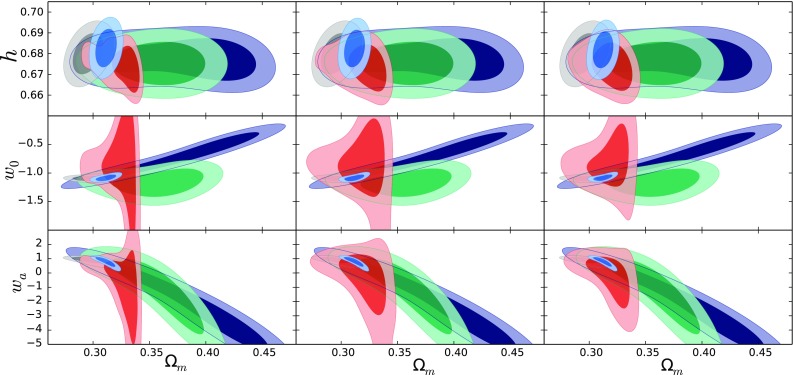
Lazkoz–Sendra pivotal model; solid contours limit $1\sigma$ regions and clear contours $2\sigma$ region. Purple for the BAO, green for SNe and red for SL data set, gray for SNe + BAO and blue SL + BAO + SNe. First set (left) for 24 years, second (middle) for 28 and third (right) for 32 years

## Appendix A: Results for the $\varLambda$CDM model

See Table 2 and Fig. 4.

**Table 2:** Parameter results of the $\varLambda$CDM model

Data set	*h*	${\varOmega _m}$	${\varOmega _b}$	${\chi ^2_\mathrm{min}}$	${\chi ^2_\mathrm{red}}$
BAO	$0.689_{-0.002}^{+0.002}$	$0.335^{+0.008}_{-0.008}$	$0.0467^{+0.0004}_{-0.0004}$	9.47	0.861
SNe	$0.675_{-0.007}^{+0.006}$	$0.301^{+0.008}_{-0.008}$		1387.41	0.510
SNe + BAO	$0.689_{-0.002}^{+0.002}$	$0.324_{-0.006}^{+0.006}$	$0.0472_{-0.0004}^{+0.0004}$	1402.07	0.513
SL (24 years)	$0.674_{-0.006}^{+0.006}$	$0.328^{+0.003}_{-0.003}$	$0.0467^{+0.0004}_{-0.0004}$	14.51	0.538
SNe + BAO + SL (24 years)	$0.689_{-0.002}^{+0.002}$	$0.324^{+0.003}_{-0.002}$		1417.82	0.513
SL (28 years)	$0.673_{-0.006}^{+0.006}$	$0.328^{+0.003}_{-0.003}$	$0.0467^{+0.0004}_{-0.0004}$	19.73	0.731
SNe + BAO + SL (28 years)	$0.689_{-0.002}^{+0.002}$	$0.325^{+0.002}_{-0.002}$		1423.49	0.515
SL (32 years)	$0.673_{-0.006}^{+0.006}$	$0.328^{+0.002}_{-0.002}$	$0.0468^{+0.0004}_{-0.0004}$	25.75	0.954
SNe + BAO + SL (32 years)	$0.689_{-0.002}^{+0.002}$	$0.325^{+0.002}_{-0.002}$		1430.03	0.518

## Appendix B: Results for the Quiessence model

See Table 3 and Fig. 5.

**Table 3:** Parameter results of the quiessence model

Data set	*h*	${\varOmega _m}$	${\varOmega _b}$	*w*	${\chi ^2_\mathrm{min}}$	${\chi ^2_\mathrm{red}}$
BAO	$0.677_{-0.006}^{+0.006}$	$0.336^{+0.008}_{-0.008}$	$0.0485^{+0.0010}_{-0.0009}$	$-0.948^{+0.024}_{-0.025}$	5.10	0.510
SNe	$0.675_{-0.007}^{+0.006}$	$0.341^{+0.013}_{-0.015}$		$-1.244^{+0.123}_{-0.122}$	1383.13	0.509
SNe + BAO	$0.686_{-0.006}^{+0.006}$	$0.323_{-0.006}^{+0.006}$	$0.0472_{-0.0008}^{+0.0008}$	$-0.987_{-0.023}^{+0.022}$	1401.74	0.513
SL (24 years)	$0.674_{-0.007}^{+0.006}$	$0.323^{+0.011}_{-0.011}$	$0.0474^{+0.0008}_{-0.0008}$	$-0.888^{+0.141}_{-0.660}$	12.73	0.490
SNe + BAO + SL (24 years)	$0.685_{-0.005}^{+0.005}$	$0.324^{+0.003}_{-0.002}$		$-0.982^{+0.020}_{-0.021}$	1417.07	0.513
SL (28 years)	$0.674_{-0.007}^{+0.006}$	$0.321^{+0.009}_{-0.009}$	$0.0475^{+0.0008}_{-0.0008}$	$-0.845^{+0.102}_{-0.176}$	17.32	0.666
SNe + BAO + SL (28 years)	$0.684_{-0.005}^{+0.005}$	$0.325^{+0.002}_{-0.002}$		$-0.979^{+0.020}_{-0.021}$	1422.49	0.515
SL (32 years)	$0.674_{-0.006}^{+0.007}$	$0.320^{+0.007}_{-0.008}$	$0.0476^{+0.0008}_{-0.0008}$	$-0.830^{+0.084}_{-0.119}$	22.62	0.870
SNe + BAO + SL (32 years)	$0.684_{-0.005}^{+0.005}$	$0.325^{+0.002}_{-0.002}$		$-0.977^{+0.020}_{-0.020}$	1428.75	0.517

## Appendix C: Results for the slow-roll model

See Table 4 and Fig. 6.

**Table 4:** Parameter results of the slow-roll model

Data set	*h*	${\varOmega _m}$	${\varOmega _b}$	${\delta w}$	${\chi ^2_\mathrm{min}}$	${\chi ^2_\mathrm{red}}$
BAO	$0.678_{-0.006}^{+0.006}$	$0.341_{-0.008}^{+0.009}$	$0.0485_{-0.0009}^{+0.0010}$	$0.074_{-0.035}^{+0.036}$	5.20	0.520
SNe	$0.675_{-0.006}^{+0.006}$	$0.330_{-0.012}^{+0.010}$		$-0.260_{-0.138}^{+0.128}$	1383.10	0.509
SNe + BAO	$0.688_{-0.006}^{+0.005}$	$0.324_{-0.006}^{+0.006}$	$0.0468_{-0.0008}^{+0.0008}$	$0.005_{-0.030}^{+0.030}$	1402.05	0.513
SL (24 years)	$0.674_{-0.006}^{+0.006}$	$0.324_{-0.007}^{+0.007}$	$0.0471_{-0.0008}^{+0.0008}$	$0.231_{-0.539}^{+0.252}$	13.12	0.504
SNe + BAO + SL (24 years)	$0.687_{-0.005}^{+0.005}$	$0.325_{-0.002}^{+0.002}$		$0.014_{-0.029}^{+0.028}$	1417.62	0.513
SL (28 years)	$0.674_{-0.006}^{+0.006}$	$0.323_{-0.006}^{+0.006}$	$0.0472_{-0.0008}^{+0.0008}$	$0.282_{-0.330}^{+0.199}$	17.85	0.686
SNe + BAO + SL (28 years)	$0.686_{-0.005}^{+0.005}$	$0.325_{-0.002}^{+0.002}$		$0.017_{-0.027}^{+0.028}$	1423.21	0.515
SL (32 years)	$0.674_{-0.006}^{+0.006}$	$0.323_{-0.005}^{+0.005}$	$0.0473_{-0.0007}^{+0.0008}$	$0.306_{-0.254}^{+0.171}$	23.31	0.897
SNe + BAO + SL (32 years)	$0.686_{-0.005}^{+0.005}$	$0.325_{-0.002}^{+0.002}$		$0.021_{-0.029}^{+0.029}$	1429.59	0.518

## Appendix D: Results for the CPL model

See Table 5 and Fig. 7.

**Table 5:** Parameter results of the CPL model

Data set	*h*	${\varOmega _m}$	${\varOmega _b}$	${w_0}$	${w_a}$	${\chi ^2_\mathrm{min}}$	${\chi ^2_\mathrm{red}}$
BAO	$0.677_{-0.007}^{+0.006}$	$0.395^{+0.035}_{-0.075}$	$0.0486^{+0.0010}_{-0.0010}$	$-0.558^{+0.311}_{-0.467}$	$-1.722^{+2.035}_{-1.457}$	3.37	0.374
SNe	$0.675_{-0.006}^{+0.006}$	$0.356^{+0.030}_{-0.038}$		$-1.165^{+0.203}_{-0.182}$	$-0.555^{+1.232}_{-1.708}$	1383.07	0.509
SNe + BAO	$0.678_{-0.006}^{+0.006}$	$0.281^{+0.011}_{-0.011}$	$0.0484^{+0.0010}_{-0.0009}$	$-1.187^{+0.040}_{-0.032}$	$1.022^{+0.119}_{-0.195}$	1386.45	0.508
SL (24 years)	$0.674_{-0.006}^{+0.007}$	$0.328^{+0.008}_{-0.017}$	$0.0476_{-0.0008}^{+0.0009}$	$-1.026^{+0.498}_{-1.518}$	$-0.001^{+1.044}_{-1.584}$	12.19	0.488
SNe + BAO+SL (24 years)	$0.684_{-0.006}^{+0.006}$	$0.314_{-0.008}^{+0.006}$		$-1.117_{-0.066}^{+0.066}$	$0.602_{-0.286}^{+0.286}$	1412.27	0.512
SL (28 years)	$0.674_{-0.006}^{+0.006}$	$0.324^{+0.010}_{-0.017}$	$0.0476_{-0.0008}^{+0.0009}$	$-0.937^{+0.423}_{-0.627}$	$-0.021^{+0.869}_{-1.541}$	16.56	0.662
SNe + BAO + SL (28 years)	$0.683_{-0.006}^{+0.006}$	$0.315_{-0.008}^{+0.006}$		$-1.113_{-0.068}^{+0.066}$	$0.590_{-0.276}^{+0.293}$	1417.52	0.513
SL (32 years)	$0.674_{-0.006}^{+0.006}$	$0.319^{+0.010}_{-0.019}$	$0.0477_{-0.0008}^{+0.0009}$	$-0.895^{+0.372}_{-0.256}$	$0.240^{+0.637}_{-1.147}$	21.65	0.866
SNe + BAO + SL (32 years)	$0.683_{-0.006}^{+0.006}$	$0.315_{-0.008}^{+0.006}$		$-1.120_{-0.062}^{+0.066}$	$0.628_{-0.278}^{+0.297}$	1423.36	0.516

## Appendix E: Results for the Lazkoz–Sendra pivotal Dark Energy model

See Table 6 and Fig. 8.

**Table 6:** Parameter results of the Lazkoz–Sendra pivotal model

Data set	*h*	${\varOmega _m}$	${\varOmega _b}$	${w_0}$	${w_a}$	${\chi ^2_\mathrm{min}}$	${\chi ^2_\mathrm{red}}$
BAO	$0.677_{-0.006}^{+0.006}$	$0.391_{-0.065}^{+0.034}$	$0.0485_{-0.0009}^{+0.0009}$	$-0.661_{-0.333}^{+0.244}$	$-2.114_{-1.820}^{+2.401}$	3.33	0.370
SNe	$0.675_{-0.007}^{+0.006}$	$0.361_{-0.031}^{+0.023}$		$-1.170_{-0.143}^{+0.150}$	$-1.063_{-2.004}^{+1.565}$	1383.13	0.509
SNe + BAO	$0.680_{-0.006}^{+0.006}$	$0.295_{-0.008}^{+0.009}$	$0.0481_{-0.0009}^{+0.0009}$	$-1.093_{-0.025}^{+0.028}$	$0.934_{-0.196}^{+0.112}$	1388.46	0.508
SL (24 years)	$0.673_{-0.006}^{+0.007}$	$0.331_{-0.014}^{+0.005}$	$0.0477_{-0.0008}^{+0.0008}$	$-1.056_{-2.390}^{+0.510}$	$-0.616_{-2.186}^{+1.416}$	12.53	0.501
SNe + BAO + SL (24 years)	$0.683_{-0.006}^{+0.006}$	$0.311_{-0.006}^{+0.006}$		$-1.088_{-0.029}^{+0.037}$	$0.826_{-0.278}^{+0.185}$	1408.54	0.510
SL (28 years)	$0.674_{-0.007}^{+0.007}$	$0.325_{-0.015}^{+0.009}$	$0.0477_{-0.0008}^{+0.0009}$	$-0.884_{-0.449}^{+0.349}$	$-0.173_{-1.775}^{+0.917}$	16.77	0.671
SNe + BAO + SL (28 years)	$0.682_{-0.006}^{+0.005}$	$0.312_{-0.006}^{+0.006}$		$-1.089_{-0.029}^{+0.037}$	$0.839_{-0.279}^{+0.177}$	1413.75	0.512
SL (32 years)	$0.674_{-0.006}^{+0.006}$	$0.322_{-0.015}^{+0.009}$	$0.0479_{-0.0008}^{+0.0008}$	$-0.848_{-0.227}^{+0.335}$	$-0.080_{-1.498}^{+0.815}$	21.93	0.877
SNe + BAO + SL (32 years)	$0.682_{-0.005}^{+0.005}$	$0.312_{-0.006}^{+0.005}$		$-1.087_{-0.028}^{+0.038}$	$0.835_{-0.274}^{+0.177}$	1419.53	0.514

## Footnotes

1 As the major axis of the contours are typically aligned with the axes of each parameters in the parameter space.
